# Nano-Heterojunction NO_2_ Gas Sensor Based on *n*-ZnO Nanorods/*p*-NiO Nanoparticles Under UV Illumination at Room Temperature

**DOI:** 10.3390/nano15181426

**Published:** 2025-09-16

**Authors:** Yoon-Seo Park, Sohyeon Kim, Junyoung Lee, Jae-Hoon Jeong, Sung-Yun Byun, Jiyoon Shin, Il-Kyu Park, Kyoung-Kook Kim

**Affiliations:** 1 Department of IT Semiconductor Convergence Engineering, Research Institute of Advanced Convergence Technology, Tech University of Korea, Siheung 15073, Republic of Korea; 2 School of Mechanical and Aerospace Engineering, Nanyang Technological University, Singapore 639798, Singapore; 3 Department of Materials Science and Engineering, Seoul National University of Science and Technology, Seoul 01811, Republic of Korea; 4 Department of Semiconductor Engineering, Tech University of Korea, Siheung 15073, Republic of Korea

**Keywords:** NO_2_ gas sensor, room-temperature detection, UV activation, nano-heterojunction, high response

## Abstract

Room-temperature (RT) gas sensors for nitrogen dioxide (NO_2_) detection face persistent challenges, including reliance on high operating temperatures and inefficient charge carrier utilization under UV activation. To address these limitations, we engineered a *p*-*n* nano-heterojunction (NHJ) gas sensor by integrating *p*-type nickel oxide (NiO) nanoparticles onto *n*-type zinc oxide (ZnO) nanorods. This architecture leverages UV-driven carrier generation and interfacial electric fields at the NHJ to suppress recombination, enabling unprecedented RT performance. By optimizing thermal annealing conditions, we achieved a well-defined heterojunction with uniform NiO distribution on the top of the ZnO nanorods, validated through electron microscopy and X-ray photoelectron spectroscopy. The resulting sensor exhibits a 5.4-fold higher normalized response to 50 ppm NO_2_ under 365 nm UV illumination compared to pristine ZnO, alongside rapid recovery and stable cyclability. The synergistic combination of UV-assisted carrier generation and heterojunction-driven interfacial modulation offers a promising direction for next-generation RT gas sensors aimed at environmental monitoring.

## 1. Introduction

Nitrogen dioxide (NO_2_), a byproduct of fossil fuel combustion, significantly contributes to air pollution. Through interactions with other pollutants, water, and photochemical reactions, NO_2_ adversely affects the environment by contributing to acid rain and smog formation. Even at concentrations in the parts-per-million (ppm) range, NO_2_ can cause respiratory illnesses when inhaled, posing severe health risks to humans. With the ongoing trends of industrialization and population growth, the global reliance on fossil fuels continues to increase, further exacerbating NO_2_ emissions. Consequently, there is a growing demand for efficient gas sensors capable of rapidly detecting low-level NO_2_ exposure. Various research groups have been actively pursuing advancements in gas-sensing technology to address this critical need [1,2,3].

Gas sensors employ a variety of detection mechanisms depending on their underlying principles. Among these, metal oxide semiconductor (MOS)-based sensors, such as those utilizing SnO_2_, In_2_O_3_, TiO_2_, CeO_2_, ZnO, and CuO, have gained significant attention due to their relatively low manufacturing costs and high gas sensitivity [4,5,6,7]. In particular, ZnO is promising for gas sensing applications due to its chemical stability, wide band gap (3.37 eV), and high exciton binding energy (~60 meV). Additionally, the unique properties of ZnO enable the fabrication of various nanostructures, including nanorods (NRs), nanowires, nanoflowers, nanotubes, and nanowalls, all of which provide large surface areas that enhance gas sensing performance [8,9,10]. Despite these advantages, gas sensors based on MOS, including ZnO, often exhibit limited response at room temperature (RT) and typically require high operating temperatures (>100 °C) to achieve reliable performance [11].

Despite these advantages, gas sensors based on MOS, including ZnO, often exhibit limited response at room temperature (RT) and typically require high operating temperatures (>100 °C) to achieve reliable performance [11]. The high-temperature operation, however, introduces several challenges, such as decreased gas selectivity, reduced long-term stability, shorter sensor lifespan, and increased power consumption.

Recent research has focused on enhancing RT gas detection performance to address these challenges. One promising approach involves utilizing the photoactivation effect, where ultraviolet (UV) light improves gas sensing at RT [12,13,14,15]. For instance, Z. Cai et al. demonstrated enhanced NO_2_ detection at RT by incorporating gold nanoparticles (NPs) into ZnO nanowires and activating the sensor using UV light [16]. Similarly, B.P.J. de Lacy Costello et al. explored the gas sensing characteristics of ZnO-based sensors using a 400 nm UV light-emitting diode (UV-LED), focusing on gases such as toluene, acetone, and pentane [17]. These studies demonstrated the potential of UV photoactivation to enhance gas sensing performance at room temperature. Building on this foundation, our previous works demonstrated the RT operation of a ZnO NR-based gas sensor utilizing UV photonic energy as a substitute for thermal energy [18,19,20]. However, despite the benefits of UV-photoactivation, this method faces limitations due to the rapid recombination of photogenerated charge carriers, which restricts the full utilization of the material’s photocatalytic properties [21,22].

To mitigate the charge carrier recombination issue, forming a *p*-*n* heterojunction can be a promising solution. A *p*-*n* heterojunction suppresses charge carrier recombination, extending the carrier lifetime and allowing more efficient photogenerated carrier use. The *p*-*n* heterostructures maintain high response when gas molecules are adsorbed onto the sensor’s surface, enabling effective detection of a wide range of harmful gases, even at low concentrations [23,24].

In this study, we overcome the limitations of conventional UV-photoactivated sensors with a novel *p*-*n* nano-heterojunction (NHJ) structure integrating *p*-type NiO onto *n*-type ZnO. NiO is widely recognized as a *p*-type oxide semiconductor, primarily attributed to nickel vacancies that introduce hole carriers [25]. This heterojunction structure of NiO NPs-supported ZnO NRs (ZnO NRs/NiO NPs) promotes effective charge carrier separation, reducing recombination losses and improving sensor response. In addition, such heterojunctions can operate efficiently at RT, an advantage over structures requiring additional thermal or surface plasmon activation. Incorporating *p*-NiO provides better structural and thermal stability, which is crucial for long-term sensor operation, especially under varying environmental conditions. This work demonstrated a significant enhancement in gas sensing performance, with sensors annealed at 800 °C exhibiting an exceptional normalized response that was 5.5 times higher than that of pristine ZnO NRs.

## 2. Materials and Methods

### 2.1. Fabrication of Sensing Materials

#### 2.1.1. Hydrothermal Synthesis of ZnO Nanorods

A ZnO seed layer was deposited on a sapphire substrate using the sol–gel method. The sol–gel solution consisted of 4.4 g of zinc acetate dihydrate (Zn(CH_3_COO)_2_∙2H_2_O), 100 mL of 2-methoxyethanol (2-ME), and 1.22 mL of monoethanolamine (MEA). The mixture was stirred and heated at 60 °C for 2 h, then stabilized at RT for 24 h. The prepared solution was then spin-coated onto the cleaned sapphire substrate. The coated substrate was annealed on a hot plate at 150 °C for 10 min to remove residual solvent. Finally, the substrate was annealed in a box furnace at 500 °C for 2 h in ambient air.

**Figure 1:**
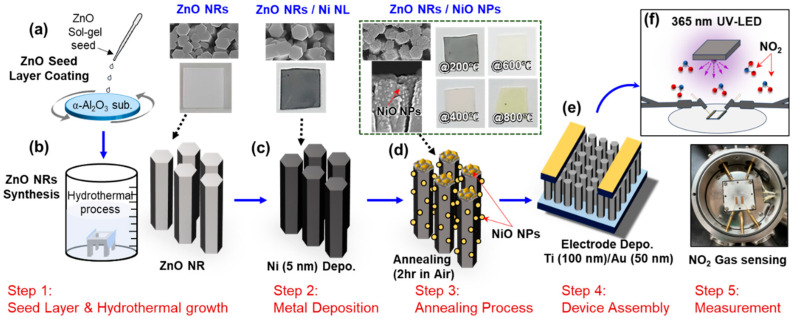
Fabrication procedure for *p*-*n* NHJ gas sensors. Schematic images of (**a**) ZnO seed layer grown on sapphire substrate, (**b**) ZnO NRs synthesized by hydrothermal method, (**c**) ZnO NRs deposited 5 nm-thick Ni layer, and (**d**) ZnO NRs/NiO NPs-based gas sensor fabricated by thermal annealing at different temperatures. (**e**) After the fabrication of the nanostructured sensing materials, electrodes were formed at both ends of the sensor. (**f**) An image of the sensor during NO_2_ detection under UV-LED illumination and a photograph of the actual gas sensing chamber.

For hydrothermal synthesis, a solution of 35 mM zinc nitrate hexahydrate and 7.5 mM hexamethylenetetramine was prepared in 300 mL of deionized water. The solution was stirred and heated to 90 °C. The sol–gel-coated sapphire substrate was then secured in a Teflon jig and immersed in the preheated solution, maintaining a constant temperature of 90 °C. The synthesis was carried out for 4 h, as shown in Figure 1a,b.

#### 2.1.2. Formation of *p*-NiO NPs/*n*-ZnO NRs Heterojunctions

A 5 nm Ni nanolayer (NL) was deposited onto the ZnO NRs using an electron beam evaporator (Telemark, Fremont, CA, USA), as shown in Figure 1c. The base pressure was maintained at 5.6 × 10^−7^ Torr, with a working pressure of 3.2 × 10^−6^ Torr. Then, heat treatment was performed in a box furnace under atmospheric air. As shown in Figure 1d, the ZnO NRs with a 5 nm thick Ni NL (ZnO NRs/Ni NL) were annealed at four different temperatures—200, 400, 600, and 800 °C—for 2 h. After annealing, the Ni layer transformed into NPs, and for simplicity, the resulting sensors are referred to as ZnO NRs/NiO NPs@200, ZnO NRs/NiO NPs@400, ZnO NRs/NiO NPs@600, and ZnO NRs/NiO NPs@800, respectively. Additionally, a pristine ZnO NRs sensor was subjected to heat treatment at different temperatures under the same conditions for comparison with the ZnO NRs/NiO NPs sensor.

#### 2.1.3. Electrode Formation

Electrodes composed of titanium (Ti, 100 nm) and gold (Au, 50 nm) were deposited using an electron beam evaporator, as shown in Figure 1e.

### 2.2. Characterizations

The structural characteristics of the gas sensors were examined using a high-resolution field emission scanning electron microscope (HR FE-SEM, Nova NanoSEM 450, FEI Company, Hillsboro, OR, USA) and a high-resolution transmission electron microscope (HR-TEM, JEM-2100F, JEOL Ltd., Tokyo, Japan). The chemical states of the materials were determined using X-ray photoelectron spectroscopy (XPS, Kratos AXIS Supra, Kratos Analytical, Manchester, UK).

### 2.3. Gas Sensing Measurements

The gas-sensing characteristics of the sensors were measured by detecting changes in electrical resistance. The normalized responsivity, R, was calculated as the ratio of the resistance in NO_2_ gas (R_g_) to that in air (R_a_): (R_g_ − R_a_)/R_a_. A data acquisition system (Keithley 2700, Tektronix, Beaverton, OR, USA) was used to monitor the sensor response. The sensors were tested under NO_2_ gas concentrations of 10 ppm, 25 ppm, and 50 ppm using a custom-built gas flow control system and a UV LED with a wavelength of 365 nm, as shown in Figure 1f. The UV LED wavelength was selected considering the absorption ranges of ZnO and NiO, and the power was maintained at 540 mW during gas sensing measurements.

## 3. Results and Discussion

### 3.1. Material Characterizations

**Figure 2:**
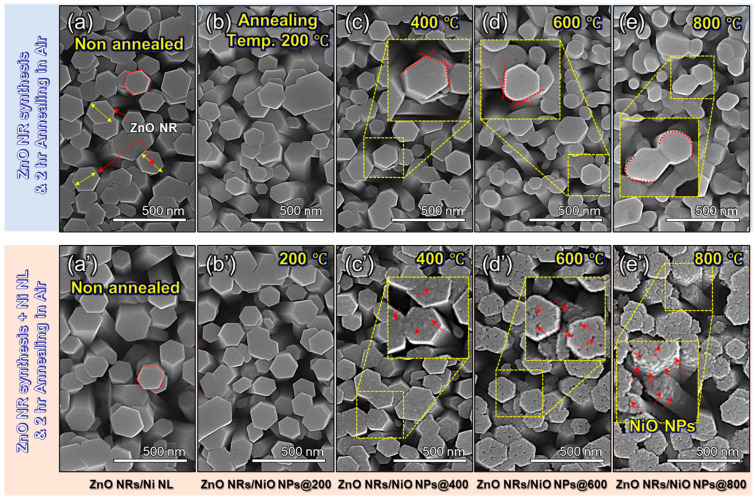
Top view FE-SEM images of (**a**) ZnO NRs synthesized by hydrothermal process and Zn NRs annealed at (**b**) 200, (**c**) 400, (**d**) 600, and (**e**) 800 °C. The ZnO NRs exhibit a vertically aligned growth with a well-defined hexagonal shape. After annealing, it was observed that the hexagonal morphology gradually became less distinct as the annealing temperature increased. The FE-SEM top view images of (**a’**) ZnO NRs deposited 5 nm thick Ni NL, (**b’**) ZnO NRs/NiO NPs@200, (**c’**) ZnO NRs/NiO NPs@400, (**d’**) ZnO NRs/NiO NPs@600, and (**e’**) ZnO NRs/NiO NPs@800 gas sensors, which were fabricated by annealing ZnO NRs/Ni NL at 200, 400, 600, and 800 °C, respectively. In the case where NiO NPs were formed on the surface of ZnO, no significant morphological changes were observed with increasing annealing temperature. In Figures (**c’**–**e’**), the red arrows indicate the morphology of the NiO NPs formed on the ZnO surface.

To examine the effect of annealing temperature on the morphology of NiO NPs on ZnO NRs, Figure 2 shows FE-SEM images of ZnO NRs, ZnO NRs/Ni NL, and ZnO NRs/NiO NPs fabricated via thermal annealing at different temperatures (ZnO NRs/NiO NPs@200, 400, 600, and 800). These results demonstrate that NiO NPs are uniformly formed on the top of the vertically grown ZnO NRs. The pristine morphology of ZnO NRs is characterized first. As shown in Figure 2a, ZnO NRs exhibit vertical and hexagonal morphology with an average diameter of approximately 150 nm. Figure S1 presents the diameter distribution of 500 individual NRs, confirming an average diameter of 150 nm. Figure 2b–e show the top-view images of ZnO NRs annealed at different temperatures. As the annealing temperature increases, the ZnO NRs undergo a morphological transformation from a hexagonal to a cylindrical shape. In Figure 2a’–e’, the influence of Ni NL deposition and annealing is examined. In case of ZnO NRs/Ni NL, no significant morphological difference was observed compared to the ZnO NRs of Figure 2a. However, the morphology after thermal annealing exhibited slightly different characteristics compared to that of pristine ZnO. In the case of ZnO NRs/Ni NL, the Ni NL gradually transformed into NP form with increasing annealing temperature, as observed in Figure 2b’–e’. Interestingly, while the pristine ZnO NRs in Figure 2b–e exhibited noticeable morphological changes upon annealing, the ZnO NRs/NiO NPs maintained a relatively stable surface morphology. The observed morphological changes in ZnO NRs are attributed to a reduction in surface energy. In contrast, for ZnO NRs/Ni NL, the annealing thermal energy is believed to facilitate the formation of NiO NPs, driven by the surface energy disparity of the Ni metal. This trend was confirmed through FE-SEM tilt images of ZnO NRs/NiO NPs, as shown in Figure S2, demonstrating a progressive increase in both the size and quantity of NiO NPs with rising annealing temperatures. The NPs began to appear clearly on the ZnO NR surfaces at annealing temperatures exceeding 400 °C, with their presence becoming more pronounced as the temperature increased. FE-SEM images confirm that NiO NPs with a uniform density for the top of ZnO NRs were distinctly formed within the temperature range of 600 to 800 °C. However, the NiO NPs on the *c*-plane surface, corresponding to the top surface of the ZnO NRs, exhibited a more significant size compared to those on the side surfaces. This phenomenon is attributed to the step coverage characteristics during the Ni metal deposition process via evaporation, wherein a more significant amount of Ni metal was deposited on the top plane than on the side surfaces. Consequently, this led to the formation of larger NiO NPs on the ZnO *c*-plane surface during the subsequent heat treatment process.

**Figure 3:**
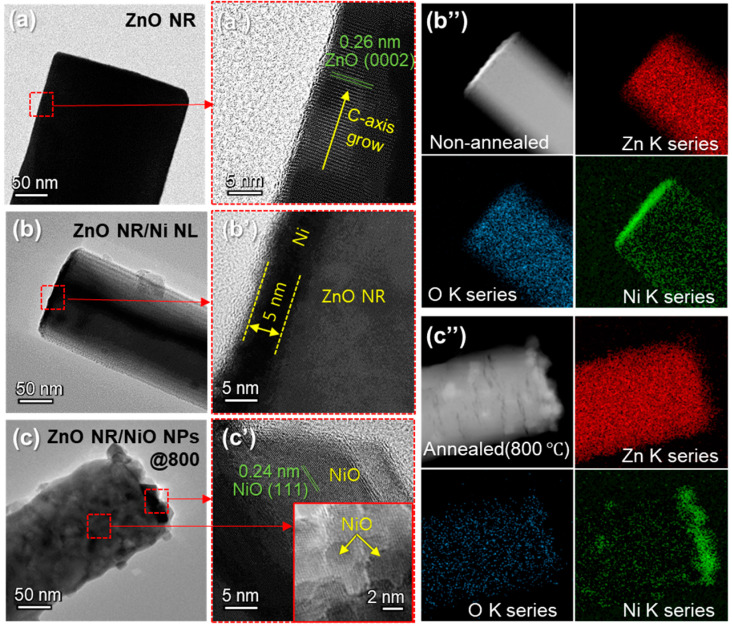
FE-TEM images of (**a**) ZnO NR, (**b**) ZnO NR/Ni NL, and (**c**) ZnO NR/NiO NPs. High-magnification FE-TEM images of (**a’**) ZnO NR, (**b’**) ZnO NR/Ni NL, and (**c’**) ZnO NR/NiO NPs. In the high-magnification FE-TEM images, the thickness of Ni NL and lattice spacings of ZnO and NiO were confirmed. EDS mapping images of (**b”**) ZnO/Ni NL and (**c”**) ZnO/NiO NPs illustrate the material transformations before and after annealing process.

To investigate the structural and compositional characteristics during NiO NP formation on ZnO NRs, high-resolution FE-TEM analysis and EDS mapping were performed. Figure 3a,a’ show that the pristine ZnO NRs exhibit a well-defined hexagonal structure with a lattice spacing of 0.26 nm, corresponding to the (0002) plane of ZnO. Upon deposition of a 5 nm thick Ni NL, the layer conformally coats the surface of ZnO NRs without altering their morphology, as shown in Figure 3b,b’. After annealing at 800 °C, significant structural changes are observed. The Ni NL transforms into NiO NPs decorating the surface of ZnO NRs (Figure 3c). Figure 3c’ shows lattice fringes of NPs with an interplanar spacing of 0.24 nm, corresponding to NiO’s (111) plane. This structural transformation leads to the formation of NHJs between ZnO and NiO, which are critical for enhanced sensing performance. Figure 3b”,c” illustrate the changes in material composition before and after annealing, as revealed through EDS mapping. Both before and after thermal annealing, Zn and O elements were uniformly distributed across the entire area of the ZnO NRs. In the case of Ni, the results highlight changes resulting from the diffusion of Ni atoms during the annealing process. Mainly, a higher concentration of Ni is observed on the top surface of the ZnO NRs. Furthermore, the EDS analysis demonstrates that the initially uniform distribution of Ni before annealing becomes non-uniform after annealing, attributed to the formation of NiO NPs.

**Figure 4:**
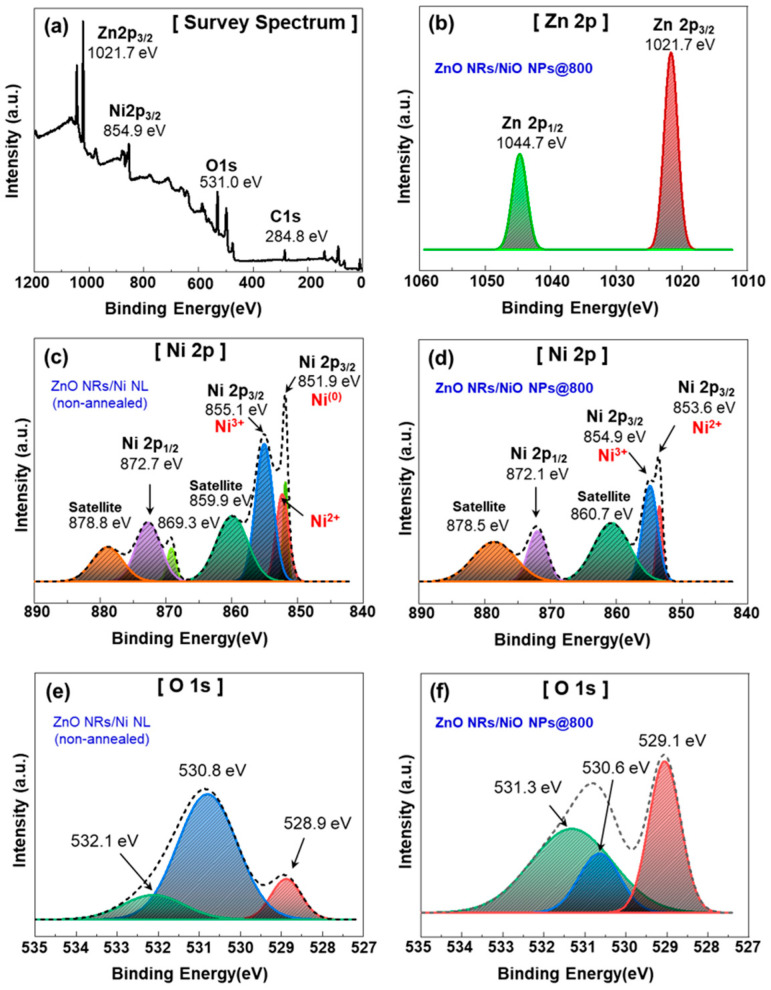
The XPS analysis was conducted to evaluate the chemical composition and oxidation states in the fabricated *p*-*n* NHJ sensor. (**a**) The survey spectrum of the presence of Zn, Ni, O, and C, with the C1s peak. (**b**) Chemical state of Zn 2p spectra for ZnO NRs/NiO NPs@800. Ni 2p spectra of (**c**) ZnO NRs/Ni NL and (**d**) ZnO NRs/NiO NPs@800. O1s spectra of (**e**) ZnO NRs/Ni NL and (**f**) ZnO NRs/NiO NPs@800. (**c**–**f**) The dashed lines represent the raw data, while the solid lines correspond to the fitting results.

Figure 4 shows that XPS analysis was conducted to evaluate the chemical composition and oxidation states in the *p*-*n* NHJ sensor, which consists of ZnO NRs/NiO NPs formed by thermal annealing of ZnO NRs/Ni NL at 800 °C. Figure 4a,b show the raw data, while in Figure 4c–f, the dashed lines represent the raw data and the solid lines correspond to the fitting results. The survey spectrum in Figure 4a confirms the presence of Zn, Ni, O, and C, with the C 1s peak at 284.8 eV used for calibration. The high-resolution spectrum in Figure 4b identifies the Zn 2p_3/2_ and Zn 2p_1/2_ peaks at 1021.7 eV and 1044.7 eV, respectively, with a spin–orbit energy separation of 23.0 eV. This value is consistent with the binding energies of pure ZnO, confirming the retention of the Zn^2+^ state in the sensor [26]. Figure 4c confirms the presence of metallic Ni^0^ in the as-deposited ZnO NRs/Ni NL. In the Ni 2p region of ZnO NRs/Ni NL, characteristic peaks appear at approximately 851.9 eV (Ni 2p_3/2_) and 869.3 eV (Ni 2p_1/2_), with a spin–orbit splitting of ~17.3 eV, which are consistent with metallic Ni^0^. Additionally, the presence of peaks at 855.1 eV (Ni 2p_3/2_), 872.7 eV (Ni 2p_1/2_), and satellite peaks at 859.9 eV and 878.8 eV suggests that some Ni has already undergone partial oxidation to form NiO before annealing [27,28]. After annealing, the disappearance of the metallic Ni^0^ peak, with only Ni^2+^ and Ni^3+^ peaks remaining, indicates the complete oxidation of Ni and the formation of *p*-type NiO. Figure 4d shows the Ni 2p region of ZnO NRs/NiO NPs obtained by annealing ZnO NRs/Ni NL at 800 °C. Peaks corresponding to Ni 2p_1/2_ and its satellite were observed at 872.1 eV and 878.5 eV, respectively. Additionally, two Ni 2p_3/2_ peaks and their satellite were detected at 853.6, 854.9, and 860.7 eV. These satellite peaks are characteristic shake-up features of Ni^2+^ in NiO, which arise from multielectron excitation and Ni 3d-O 2p charge-transfer processes [29]. Their presence in Figure 4c indicates partial oxidation of Ni, while in Figure 4d, they confirm the complete conversion of metallic Ni into NiO. Unlike in Figure 4c, a notable feature after annealing is the absence of the metallic Ni^0^ peak. Instead, only a Ni^2+^ peak was observed at 853.6 eV (Ni 2p_3/2_), confirming that metallic Ni was oxidized to NiO. Furthermore, the peak near 855 eV indicates the presence of Ni^3+^. Rather than implying the formation of Ni_2_O_3_, this suggests that NiO contains mixed Ni^2+^/Ni^3+^ states. Such a non-stoichiometric NiOx layer with mixed valence is responsible for the *p*-type conductivity of NiO. This phenomenon was observed not only after thermal annealing at 800 °C, but also at 200, 400, and 600 °C, as shown in Figure S3. Previous studies have shown that the *p*-type behavior of NiO is closely associated with the presence of Ni^2+^ and Ni^3+^ [30,31,32]. Therefore, the XPS results clearly demonstrate that the annealing process converts metallic Ni into *p*-type NiO.

To further evaluate the NHJ formation of ZnO NR and NiO NP, the O 1s spectrum of ZnO NRs/Ni NL and ZnO NRs/NiO NPs were analyzed in Figure 4e,f, respectively. In Figure 4e, the spectrum exhibits three main peaks at approximately 528.9 eV, 530.8 eV, and 532.1 eV, which are assigned to NiO lattice oxygen, ZnO lattice oxygen or oxygen-related defect states, and surface-adsorbed oxygen species, respectively [33]. After annealing, as shown in Figure 4f, the NiO-related signal (529.1 eV) increases markedly, while the ZnO-associated signal (530.6 eV) decreases. Notably, the surface oxygen peak initially located at 532.1 eV shifts to 531.3 eV and becomes more pronounced. These results indicate that annealing promotes the oxidation of Ni into a stable, crystalline NiO phase, which partially masks the ZnO signal and modifies the surface oxygen adsorption characteristics. These results indicate that annealing promotes the oxidation of Ni into a stable, crystalline NiO phase, which partially masks the ZnO signal and alters surface oxygen adsorption characteristics. Thus, the XPS analysis confirms that metallic Ni was completely oxidized to NiO through the thermal annealing process, and that the NiO present on the surface of the ZnO NRs exhibits *p*-type behavior. Therefore, it can be concluded that the *n*-type ZnO NRs and *p*-type NiO NPs form a stable *p*-*n* NHJ structure [27,34].

### 3.2. Gas Sensing Properties

**Figure 5:**
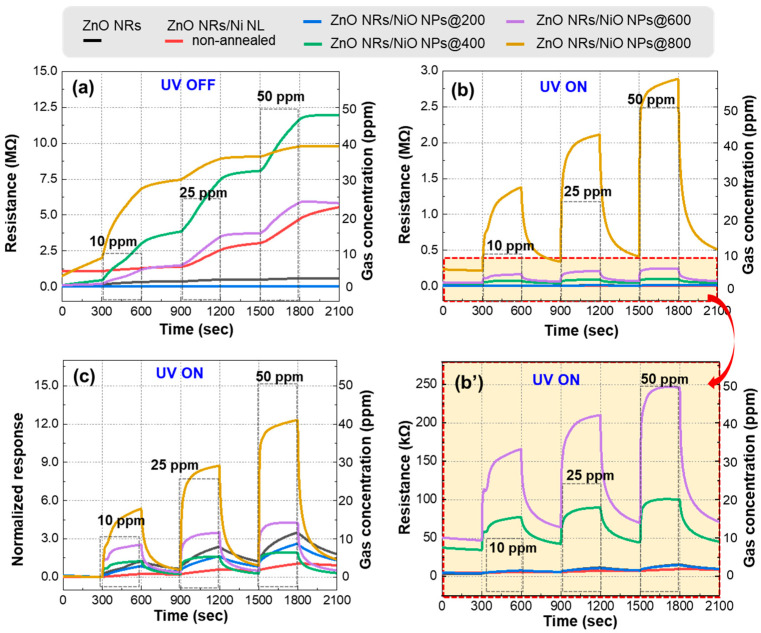
(**a**) The resistance of gas sensors at different NO_2_ gas concentrations in the dark. (**b**,**b’**) Gas sensing response of gas sensors under UV irradiation. (**c**) The normalized response of gas sensors at different NO_2_ gas concentrations under UV irradiation.

Figure 5 presents the results of NO_2_ gas sensing measurements conducted on ZnO NRs, ZnO NRs/Ni NL, and ZnO NRs/NiO NPs. The gas sensing tests were performed at RT under atmospheric pressure with NO_2_ concentrations of 10, 25, and 50 ppm. Measurements were taken in both dark and illuminated environments to evaluate the effect of UV light activation. Figure 5a shows the resistance changes for each gas sensor under various gas concentrations without UV illumination. Without UV illumination, the gas sensors exhibited negligible recovery despite showing clear sensing response. When the sensor was exposed to NO_2_, the resistance of all sensors increased steadily over time, showing gas-sensing activity. However, the recovery, where the resistance returns to its baseline after air exposure again, was almost negligible without UV light. Figure 5b,b’ show the resistance changes in each sensor when applying UV light. Under UV illumination, the ZnO NRs/NiO NPs gas sensors exhibited significantly improved recovery and response. Moreover, gas sensors annealed at higher temperatures exhibited increased base resistance values. This phenomenon may be attributed to the stronger influence of the NHJ structure at higher annealing temperatures, where the depletion region becomes more significant. Figure 5c presents the normalized response values derived from the resistance measurements from Figure 5b. Table S1 shows the detailed normalized response values of each gas sensor. In the case of ZnO NRs/NiO NPs@400 gas sensor, it showed superior recovery characteristics compared to the pristine ZnO sensor. In the case of ZnO NRs/NiO NPs@600 and ZnO NRs/NiO NPs@800, ZnO NRs/NiO NPs exhibited better sensing and recovery characteristics than the pristine ZnO gas sensor. Notably, the ZnO NRs/NiO NPs@600 sensor exhibited a normalized response 1.75 times higher than that of pristine ZnO NRs at 50 ppm NO_2_. Furthermore, the ZnO NRs/NiO NPs@800 sensor, which exhibited a-normalized response of 12.3 due to the formation of a well-defined *p*-*n* NHJ, showed a 5.4-fold higher normalized response compared to the pristine ZnO NRs sensor. As shown in Table 1, this result demonstrates a superior to NO_2_ at comparable concentrations when compared with previous studies. Notably, our sensor exhibited enhanced response at RT, suggesting that the present work provides a significant advancement in the development of RT-operable gas sensors.

**Figure 6:**
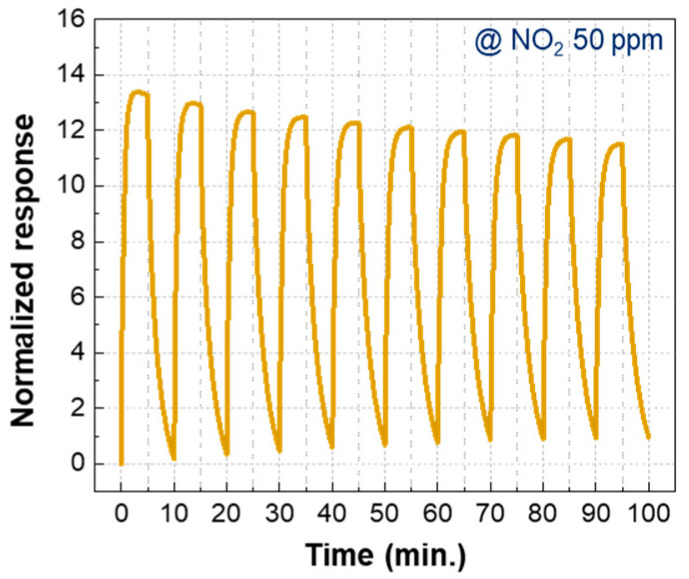
Gas sensing performance of the ZnO NRs/NiO NPs@800 sensor under 10 repeated exposures to 50 ppm NO_2_. Each cycle consisted of 5 min of NO_2_ injection followed by 5 min of air purging. A slight decrease in the normalized response was observed with increasing number of cycles.

Figure 6 shows gas sensing data for the ZnO NRs/NiO NPs@800 gas sensor to assess its reliability. The sensor’s performance over 10 repetitive tests at 50 ppm NO_2_. The normalized response exhibited a slight decline as the measurement was repeated up to 10 cycles. Nevertheless, the sensor maintained a high response level, with a normalized response exceeding 10, demonstrating its promising repeatability and potential for practical applications.

### 3.3. Gas Sensing Mechanisms

#### 3.3.1. Gas Sensing Mechanism of ZnO with UV Activation

In the case of gas sensing of ZnO without UV photonic energy, MOSs like ZnO exhibit changes in their electrical properties upon exposure to specific gases, as gas molecules adsorb onto their surface. These adsorbed gas molecules either capture or release charge, thereby altering the charge transport properties of the semiconductor, leading to observable changes in conductivity. In ambient air, oxygen molecules are chemically adsorbed onto the ZnO surface, and the environmental temperature influences the adsorption process. These adsorbed oxygen molecules capture free electrons from the ZnO conduction band, forming oxygen anions (O_2_^−^) on the surface. As a result, an electron depletion region forms, increasing the potential barrier height, hindering electron mobility, and increasing the device’s resistance. When ZnO is exposed to a NO_2_-rich environment, NO_2_ molecules chemically adsorb onto the ZnO surface and further capture electrons from the conduction band. The interaction between oxygen anions and NO_2_ molecules also causes electrons to migrate toward the NO_2_, reducing the number of surface oxygen anions. This results in an expansion of the electron depletion region and an increase in the potential barrier height, causing the sensor’s resistance to rise.

**Figure 7:**
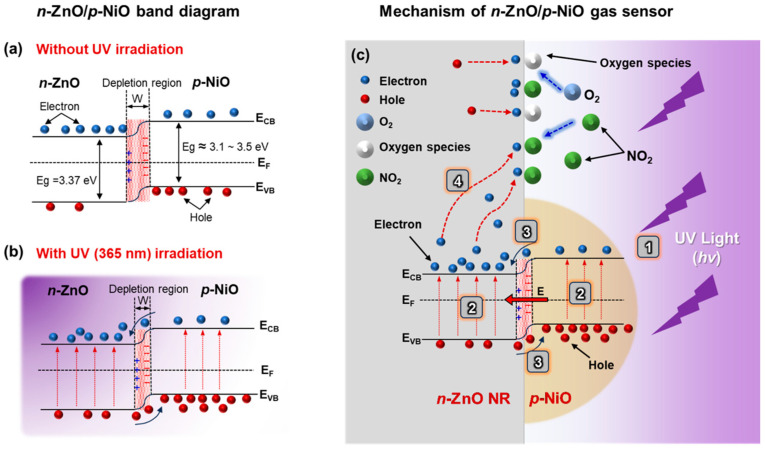
Band diagrams of ZnO NRs/NiO NPs *p*-*n* NHJ gas sensor (**a**) without UV irradiation and (**b**) with UV irradiation. (**c**) NO_2_ sensing mechanism of ZnO NRs/NiO NPs *p*-*n* NHJ gas sensor under UV irradiation at RT. The numbers represent the sequential steps of the gas-sensing mechanism: (1) UV light is irradiated onto the *p*-*n* NHJ gas sensor. (2) Photo-induced carriers are generated on the surfaces of ZnO and NiO, respectively. (3) The built-in electric field at the ZnO/NiO NHJ interface drives the separation of electrons and holes, thereby prolonging the recombination time of the electron-hole pairs. (4) The photo-induced electrons serve as additional carriers for the detection of NO_2_ gas.

On the other hand, in the case of gas sensing with UV photonic energy, ZnO generates electron-hole pairs upon exposure to UV light in ambient conditions. The UV light excites electrons, causing them to transition from the valence band to the conduction band, thus increasing the electron density in the conduction band and reducing the initial resistance of the sensor, as shown in Figure 7a,b. The photogenerated holes facilitate the desorption of oxygen ions (O_2_^−^(gas)) from the ZnO surface, contributing to the recovery process by cleaning the surface. Oxygen photo-activated species, such as O_2_^−^(hv), exhibit higher reactivity than chemically adsorbed O_2_^−^(gas), enhancing surface activity and promoting a more vigorous sensing response. When ZnO is exposed to NO_2_ gas under UV light, NO_2_ molecules, due to their higher electronegativity than oxygen molecules, further reduce the electron density in ZnO. The active photo-absorbed oxygen ions (O_2_^−^(hv)) react with NO_2_ to form NO_2_^−^ ions, which leads to an overall increase in electrical resistance. Upon returning the sensor to ambient conditions, the photogenerated holes assist in desorbing NO_2_ molecules, facilitating the sensor’s recovery process [40,41].

#### 3.3.2. UV Photoactivation in *n*-ZnO/*p*-NiO NHJs

The activation of gas sensors with UV photonic energy at RT is effective when the UV wavelength is shorter than the material’s bandgap, because this excites electrons and creates electron-hole pairs, inducing a photoactivation effect. UV photonic energy, such as UV light, generates a photocurrent on the sensor’s surface, increasing the electron density in the conduction band and lowering the sensor’s initial resistance. Furthermore, as mentioned in Section 3.3.1, oxygen species absorbed under UV exposure exhibit higher reactivity than those adsorbed under normal conditions, leading to enhanced surface activity.

However, the limitation of UV photoactivation lies in the rapid recombination of photogenerated charge carriers, which restricts the catalytic function and diminishes the optimal utilization of photocatalytic properties. To address this limitation, a *p*-*n* NHJ can be employed to suppress charge recombination and extend the lifetime of the photogenerated charge carriers, thereby allowing for more efficient utilization of these charges. In a *p*-*n* NHJ structure, an internal electric field forms at equilibrium, creating a negative charge in the *p*-type NiO and a positive charge in the *n*-type ZnO. Under UV illumination, this internal electric field efficiently separates the photogenerated electron-hole pairs, with holes migrating toward the *p*-type NiO and electrons toward the *n*-type ZnO, which enhances photocatalytic activity, as shown in Figure 7c.

Several recent reports have investigated NO_2_ sensing mechanisms based on ZnO/NiO heterostructures. For instance, S. Bai et al. synthesized rGO–NiO/ZnO composites via a hydrothermal process and hydrazine hydrate reduction, achieving a normalized response of 75.3 toward 2 ppm NO_2_ at 140 °C [42]. They attributed the enhanced response primarily to the formation of an additional depletion layer at the *p*-*n* heterojunction interface. Similarly, J. Coelho Tagliaferro et al. prepared ZnO needle- and donut-like particles through a precipitation method and subsequently combined them with NiO via hydrothermal reaction to form an *n*-*p* heterojunction [43]. In their study, the response improvement was explained by the creation of a potential barrier against charge-carrier diffusion at the *n*-*p* interface, along with the presence of an extended depletion region. Furthermore, R. R. Ambi et al. reported a NiO-coated porous ZnO film that exhibited a 76.5% normalized response toward 100 ppm NO_2_ at 150 °C, where the enhancement was attributed to the formation of interfacial charge barriers at the *n*-ZnO/*p*-NiO junction, as well as the high oxygen affinity and catalytic activity of NiO [44].

The sensing mechanism observed in ZnO/NiO heterojunction-based systems is therefore consistent across prior studies, with the depletion layer and interfacial barrier effects playing central roles. However, while our results also rely on the same fundamental heterojunction mechanism reported in previous studies, the present work further advances this concept by showing the synergistic interaction between the heterojunction structure and UV illumination. Specifically, we demonstrate that photo-driven carrier generation further strengthens the interfacial modulation in the *p*-*n* NHJ, thereby producing enhanced response at RT. This dual mechanism represents progress over conventional heterojunction-based sensors and gives a clearer understanding of charge behavior during UV-assisted sensing. Consequently, the *p*-*n* NHJ structure significantly improves gas sensors’ performance under UV light, providing a strategic advantage in RT gas sensing technologies.

## 4. Conclusions

The ZnO/NiO NHJ sensor developed here addressed critical limitations in RT NO_2_ detection by synergizing UV activation with interfacial charge engineering. Hydrothermal synthesis of ZnO NRs, followed by controlled Ni NL deposition and thermal annealing, enabled precise formation of *p*-type NiO NPs on *n*-type ZnO NRs, as validated by XPS and electron microscopy. The NiO NPs formed after annealing at 800 °C yield a well-defined heterojunction with minimal charge recombination and enhanced surface oxygen adsorption. This structural optimization resulted in a 5.4-fold increase in the normalized response to 50 ppm NO_2_ under UV compared to pristine ZnO NRs, alongside rapid recovery (<5 min) and stable response values over 10 cycles. The dual mechanism—UV-driven carrier generation and heterojunction-induced charge separation—effectively overcomes recombination losses, enabling robust RT operation without external heating. This work underscores the potential of *p*-*n* NHJ engineering in advancing low-power and high-performance gas sensors for environmental monitoring and scalable nanomaterial-based sensing platforms.

## Figures and Tables

**Table 1:** Comparison of the structure and performance of NO_2_ gas sensors.

Material	Operating Temp. (°C)	NO_2_ Concentration (ppm)	Normalized Response	UV	Ref.
CuO/ZnO Heterostructure	150	50	0.7	X	[35]
1.5 at % Ni-doped ZnO film	200	100	4.82	X	[36]
Cu-doped ZnO	175	60	5.82	X	[37]
ZnO nanowire /CuO nanoparticle	150	50	0.89	X	[38]
ZnO nanorod/Porous Silicon Nanowire	RT	50	0.35	X	[39]
ZnO nanorod /NiO nanoparticle	RT	50	12.3	O	This Work

## Data Availability

The original contributions presented in this study are included in the article/supplementary material. Further inquiries can be directed to the corresponding author(s).

## Supplementary Materials

The following supporting information can be downloaded at: https://www.mdpi.com/article/10.3390/nano15181426/s1. Figure S1: Diameter distribution of fabricated ZnO NRs. The diameters of 500 individual ZnO NRs were measured, ranging from 60 to 260 nm, with an average diameter of approximately 150 nm. Figure S2: The FE-SEM tilted images of (**a**) ZnO NRs/NiO NPs@200, (**b**) ZnO NRs/NiO NPs@400, (**c**) ZnO NRs/NiO NPs@600, and (**d**) ZnO NRs/NiO NPs@800. The FE-SEM cross-sectional images of (**a’**) ZnO NRs/NiO NPs@200, (**b’**) ZnO NRs/NiO NPs@400, (**c’**) ZnO NRs/NiO NPs@600, and (**d’**) ZnO NRs/NiO NPs@800. As the annealing temperature increases, the Ni NL is transformed into NiO NPs, and the particle size is observed to increase accordingly. In the cross-sectional view, the NPs are mainly located on the upper parts of the ZnO NRs, which can be attributed to the shadow effect inherent in the physical vapor deposition (PVD) process used for Ni NL deposition. Figure S3: XPS Ni 2p spectra of (**a**) ZnO NRs/NiO NPs@200, (**b**) ZnO NRs/NiO NPs@400, and (**c**) ZnO NRs/NiO NPs@600, which reveal the presence of both Ni^2+^ and Ni^3+^ peaks, indicating that NiO exists partially in a p-type state in all type of gas sensors. Table S1: Table of the normalized response for all fabricated gas sensors, including results under both UV-on and UV-off conditions. In the UV-off condition, all fabricated gas sensors exhibited incomplete recovery behavior. As a result, the normalized response could not be reliably determined, since the resistance did not return to the baseline level after gas exposure.
