# Supplementary material for: Nano-Heterojunction NO2 Gas Sensor Based on n-ZnO Nanorods/p-NiO Nanoparticles Under UV Illumination at Room Temperature
Source: Nanomaterials (Basel). 2025 Sep 16;15(18):1426. doi: 10.3390/nano15181426 (PMC12472634; doi:10.3390/nano15181426)
Supplement: Supplementary file 1 [file nanomaterials-15-01426-s001.zip › nanomaterials-3832549-supplementary.pdf]

Supplementary Material

Nano-Heterojunction NO_2 Gas Sensor Based on *n*-ZnO Nano-rods/*p*-NiO Nanoparticles under UV Illumination at Room Temperature

Yoon-Seo Park [1,†], Sohyeon Kim [1,†], Junyoung Lee [1], Jae-Hoon Jeong [1], Sung-Yun Byun [1], Ji-yoon Shin [2], Il-Kyu Park [3] and Kyoung-Kook Kim [1,4,*]

[1] Department of IT Semiconductor Convergence Engineering, and Research Institute of Advanced Convergence Technology, Tech University of Korea, 237 Sangidaehak-ro, Siheung-si 15073, Korea
[2] School of Mechanical and Aerospace Engineering, Nanyang Technological University, 50 Nanyang Avenue, Singapore 639798, Singapore
[3] Department of Materials Science and Engineering, Seoul National University of Science and Technology, Seoul 01811, Republic of Korea
[4] Department of Semiconductor Engineering, Tech University of Korea, 237 Sangidaehak-ro, Siheung-si 15073, Korea

[†] These authors contributed equally to this work.

[*] Correspondence: kim.kk@tukorea.ac.kr

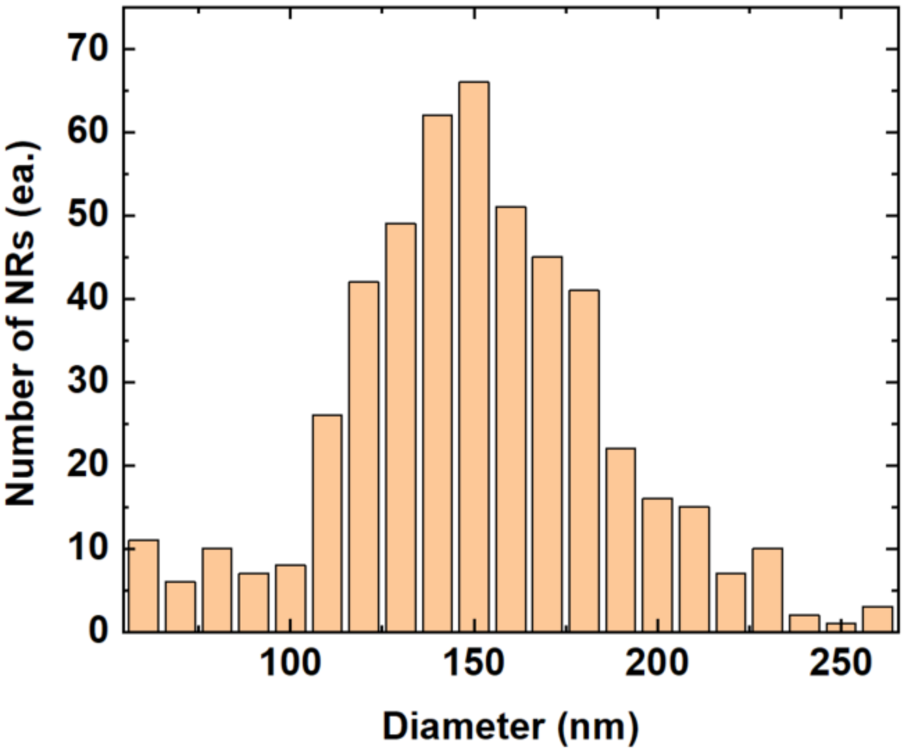

Figure S1. Diameter distribution of fabricated ZnO NRs. The diameters of 500 individual ZnO NRs were measured, ranging from 60 to 260 nm, with an average diameter of approximately 150 nm.

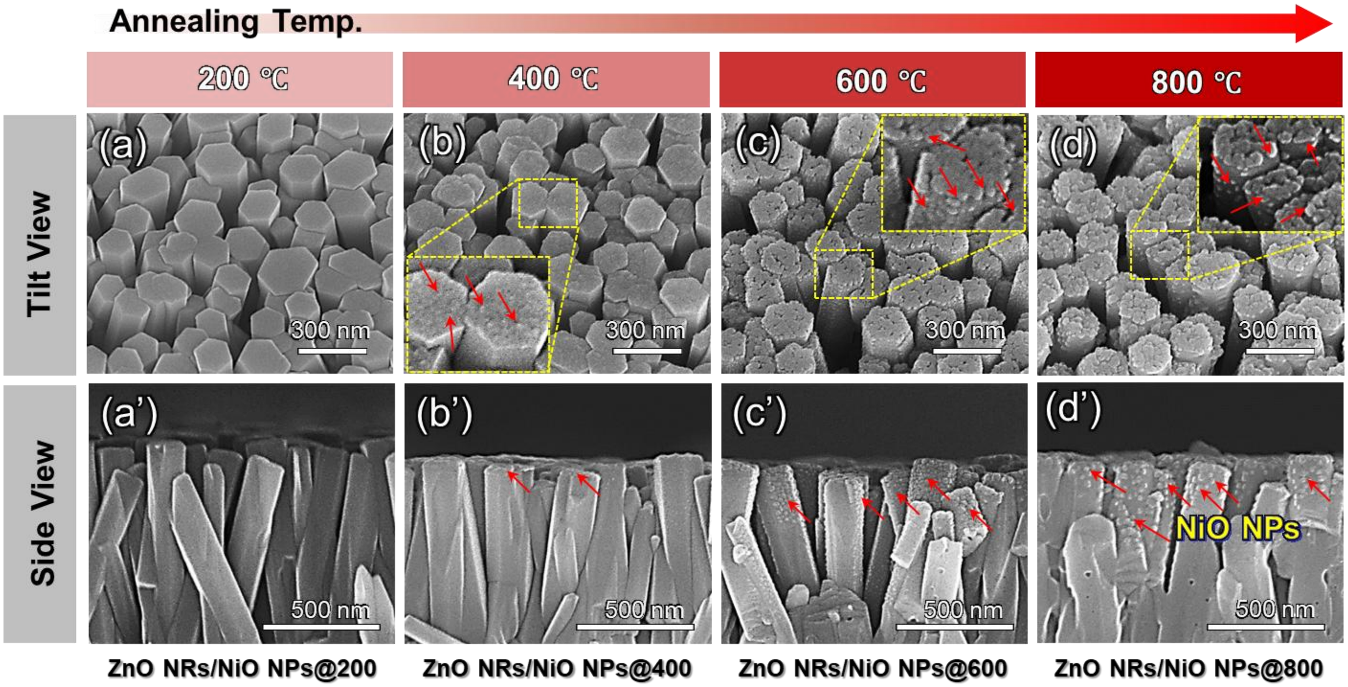

Figure S2. The FE-SEM tilted images of **(a)** ZnO NRs/NiO NPs@200, **(b)** ZnO NRs/NiO NPs@400, **(c)** ZnO NRs/NiO NPs@600, and **(d)** ZnO NRs/NiO NPs@800. The FE-SEM cross-sectional images of **(a')** ZnO NRs/NiO NPs@200, **(b')** ZnO NRs/NiO NPs@400, **(c')** ZnO NRs/NiO NPs@600, and **(d')** ZnO NRs/NiO NPs@800. As the annealing temperature increases, the Ni NL is transformed into NiO NPs, and the particle size is observed to increase accordingly. In the cross-sectional view, the NPs are mainly located on the upper parts of the ZnO NRs, which can be attributed to the shadow effect inherent in the physical vapor deposition (PVD) process used for Ni NL deposition.

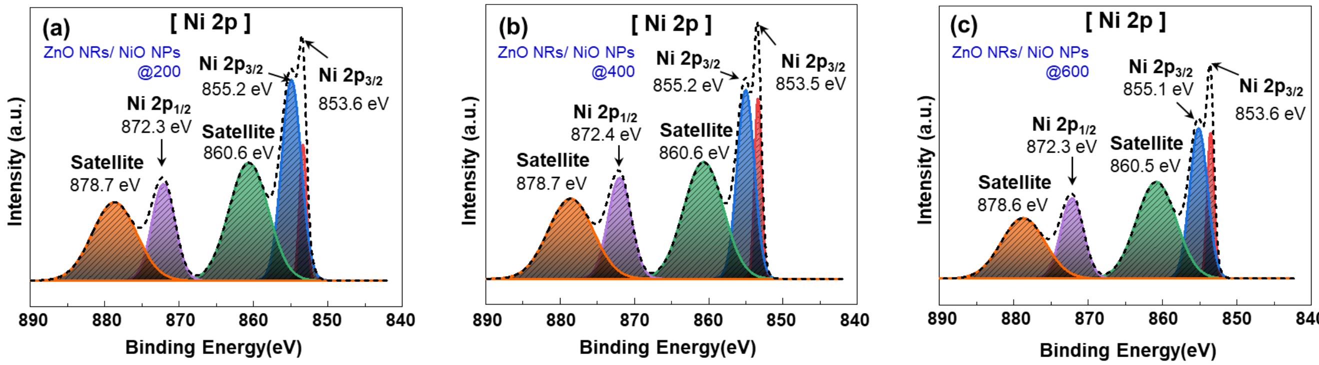

Figure S3. XPS Ni 2p spectra of **(a)** ZnO NRs/NiO NPs@200, **(b)** ZnO NRs/NiO NPs@400, and **(c)** ZnO NRs/NiO NPs@600, which reveal the presence of both Ni^{2+} and Ni^{3+} peaks, indicating that NiO exists partially in a *p*-type state in all type of gas sensors.

Table S1. Table of the normalized response for all fabricated gas sensors, including results under both UV-on and UV-off conditions. In the UV-off condition, all fabricated gas sensors exhibited incomplete recovery behavior. As a result, the normalized response could not be reliably determined, since the resistance did not return to the baseline level after gas exposure.

Sample	UV	Normalized response		
		10 ppm	25 ppm	50ppm
ZnO NRs	On	1.26	1.76	2.29
	Off	1.65	-	-
ZnO NRs/Ni NL	On	0.25	0.38	0.55
	Off	0.20	-	-
ZnO NRs/Ni NPs@200	On	0.86	1.23	1.81
	Off	0.11	-	-
ZnO NRs/Ni NPs@400	On	1.23	1.38	1.61
	Off	5.57	-	-
ZnO NRs/Ni NPs@600	On	2.54	3.12	3.73
	Off	5.08	-	-
ZnO NRs/Ni NPs@800	On	5.35	8.16	12.30
	Off	2.39	-	-
